# Evaluation of direct antiviral activity of the Deva-5 herb formulation and extracts of five Asian plants against influenza A virus H3N8

**DOI:** 10.1186/1472-6882-14-235

**Published:** 2014-07-10

**Authors:** Namsrai Oyuntsetseg, Maxim A Khasnatinov, Perenlei Molor-Erdene, Janchiv Oyunbileg, Aleksander V Liapunov, Galina A Danchinova, Sanduijav Oldokh, Jigden Baigalmaa, Chimedtseren Chimedragchaa

**Affiliations:** 1 Institute of Traditional Medicine, Mongolian National University of Medical Sciences, Zorig str, Sukhbaatar district, Ulaanbaatar, Mongolia; 2 Federal State Public Institution Scientific Centre of Family Health and Human Reproduction Problems Siberian Branch of Russian Academy of Medical Sciences, K. Marks Str. 3, Irkutsk, Russian Federation; 3 National Centre for Public Health, Ministry of Health, Peace Avenue-17, Bayanzurkh district, Ulaanbaatar, Mongolia; 4 Traditional Medical Science Technology and Production Corporation of Mongolia, Khan Uul district, Chinggis Avenue, Ulaanbaatar 17032, Mongolia

**Keywords:** *Gentiana decumbens*, *Momordica cochinchinensis*, *Hypecoum erectum*, *Polygonum bistorta*, *Terminalia chebula*, Deva-5, Influenza A virus, H3N8, Antivirals

## Abstract

**Background:**

The herb formulation Deva-5 is used in traditional medicine to treat acute infectious diseases. Deva-5 is composed of five herbs: *Gentiana decumbens* L., *Momordica cochinchinensis* L., *Hypecoum erectum* L., *Polygonum bistorta* L., and *Terminalia chebula* Retz. Deva-5 and its five components were investigated for in vitro antiviral activity against avian influenza A virus subtype H3N8.

**Methods:**

The water extracts of the herbal parts of *G. decumbens*, *H. erectum* and *P. bistorta*, the seeds of *T. chebula* and *M. cochinchinensis* and Deva-5 were prepared by boiling and clarified by low-speed centrifugation and filtration. To assess the antiviral properties, avian influenza virus isolate A/Teal/Tunka/7/2010(H3N8) was incubated at 37°C for 30 min in the presence and absence of the extracts of five plants and DEVA-5 in various concentrations. Subsequently, the concentration of infectious virus in each sample was determined by plaque assays. Neutralisation indexes and 90% plaque reduction concentrations were estimated for each extract, and the significance of the data was evaluated using statistical methods.

**Results:**

The extracts of *G. decumbens*, *H. erectum, P. bistorta* and Deva-5 demonstrated no significant toxicity at concentrations up to 2%, whereas extracts of *T. chebula* and *M. cochinchinensis* were well-tolerated by Madin-Darby canine kidney cells at concentrations up to 1%. The extracts of *H. erectum*, *M. cochinchinensis* and *T. chebula* reduced the titre of A/Teal/Tunka/7/2010 (H3N8) by approximately five-fold (p ≤ 0.05). The other three extracts did not significantly reduce the infectivity of the virus. The plaque reduction neutralisation tests revealed that none of the extracts tested were able to inhibit formation of plaques by 90%. However, three extracts, *H. erectum*, *T. chebula* and *M. cochinchinensis*, were able to inhibit formation of plaques by more than 50% at low dilutions from 1:3 to 1:14. The *T. chebula* extract had a concentration-dependent inhibitory effect.

**Conclusions:**

For the first time, the consistent direct antiviral action of the extracts of *H. erectum*, *T. chebula* and *M. cochinchinensis* was detected. These extracts significantly reduced the infectivity of influenza A virus H3N8 in vitro when used at high concentrations (0.5–1%). However, Deva-5 itself and the remainder of its components did not exhibit significant antiviral action. The results suggest that *H. erectum*, *T. chebula* and *M. cochinchinensis* plants contain substances with direct antiviral activity and could be promising sources of new antiviral drugs.

## Background

Influenza A virus (IAV) is important pathogen that causes acute disease in humans and domestic animals. In Mongolia, during the 2010/2011 influenza season, an average of 1,232 influenza-like illnesses per 10,000 people was recorded, comprising 5.8% of all recorded outpatient visits [1].

The IAV belongs to the family Orthomyxoviridae and is currently divided into subtypes according to the structure of the major virion proteins – haemagglutinin (HA) and neuraminidase (NA). To date, up to 17 subtypes of HA and up to 10 subtypes of NA have been differentiated [2]. Birds are the natural host of the virus and can harbour any subtype of IAV. However, from time to time, some avian IAVs adapt to mammalian hosts, posing a serious threat to healthcare and the economy [3]. For example, the human-avian reassortant virus H3N2 entered the human population in 1968 and is currently the major agent of seasonal flu, causing 3–5 million human cases of acute respiratory illness worldwide [3,4]. Equine IAV (H3N8) causes acute disease in horses and regularly diverges from avian IAV, most recently occurring in 1989 [5]. At present, equine IAV affects 2.1 million Mongolian horses and critically impacts the economy and nomadic livelihood in Mongolia [6].

The herb formulation Deva-5 is used in traditional medicine to treat acute infectious diseases [7]. Deva-5 is composed of five different herbs: *Gentiana decumbens* L. (26.3%), *Momordica cochinchinensis* L. (18.4%), *Hypecoum erectum* L. (15.7%), *Polygonum bistorta* L. (23.6%) and *Terminalia chebula* Retz. (15.7%) [8].

*G. decumbens* is traditionally used to cure infectious diseases and stomach disorders [9]. It has been reported to have potent anti-oxidant activity [10]. In traditional medicine, *M. cochinchinensis* is used to treat liver, stomach and kidney disorders, and it has detoxifying and antipyretic properties [9]. The anti-inflammatory, anti-oxidant, and antitumor activities of *M. cochinchinensis* have been demonstrated [11-13]. *H. erectum* is used to cure infectious disorders in traditional medicine, and it has antibacterial, antinociceptive and antipyretic properties [9]. Studies have shown antibacterial and anti-inflammatory activities of *H. erectum*[14,15]. *P. bistorta* reduces oedema and is used to treat lung disorders in traditional medicine [9]. Its anti-inflammatory effect has been reported [16]. *T. chebula* has been described in books of traditional medicine as a panacea and is used for the treatment of most diseases, especially stomach disorders [9]. *T. chebula* has been most extensively studied among the constituents of Deva-5. It has a wide spectrum of pharmacological and medicinal activities that include anti-oxidative, antibacterial, antiviral, anti-inflammatory and immunomodulatory activities [17,18]. Recently, it was shown to protect epithelial cells against damage caused by influenza A virus [19].

In the present study, we probed the direct antiviral effects of Deva-5 and its constituents against the recently isolated avian influenza virus H3N8, which is homologous in its HA structure to human H3N2 and equine H3N8 influenza viruses of epidemiological and veterinary importance.

## Methods

### Plant material and preparation of extracts

The aerial parts of *G. decumbens*, *H. erectum*, and *P. bistorta* were obtained from Tuv province in Mongolia; seeds of *M. cochinchinensis* and *T. chebula* were imported from China. The Deva-5 herb formulation and its five components were prepared in the traditional medical factory of the Traditional Medical Science Technology and Production Corporation of Mongolia. The plants were identified by a botanist at the Institute of Traditional Medicine, and voucher specimens were deposited and are publicly available at the Herbarium of Institute of Traditional Medicine, Mongolian National University of Medical Sciences (abbreviation of the herbarium, tserentsoo@mnums.edu.mn).

To minimise the effect of the preparation procedures on the bioactive compounds in the herbs, the extracts were prepared with boiling water. Briefly, 30 g, 10 g, 2 g or 1 g of each plant material was suspended in 200 ml of sterile double distilled water (ddH_2_O) and boiled at low heat for 15–30 min. When the total volume of the extract had reached a volume slightly less than 100 ml, the extracts were measured and made up to 100 ml with sterile ddH_2_O if necessary. Then, the extracts were filtered through a sterile gauze filter and finally through sterile 0.45 μm filter paper. All extraction and purification work was done aseptically and the resulting extracts were stored at +4°C for 1–2 h before the experiments.

### Cells

Madin-Darby canine kidney (MDCK) cells were purchased from BioloT (Saint Petersburg, Russian Federation). Cells were maintained at 37°C in DMEM culture media (with L-glutamine) supplemented with 10% foetal calf serum, 25 mМ HEPES and 0.2% bovine serum albumin. The cells were sub-cultured twice a week using 0.25% trypsin solution supplemented with 0.5 mM EDTA (BioloT, Saint Petersburg, Russian Federation).

### Virus maintenance and plaque titration assay

Avian influenza virus (AIV) isolate A/Teal/Tunka/7/2010(H3N8) was obtained from cloacal swabs of European teal (*Anas crecca*) sampled in eastern Siberia in 2010. The virus underwent three passages in chicken embryos and one passage in MDCK cells. The stock virus was propagated in DMEM culture media supplemented with 25 mМ HEPES, 0.2% bovine serum albumin, 2 μg/ml of TPCK-trypsin and antibiotics, then aliquoted and stored at −80°C until use [20]. Infected cell cultures were incubated at 37°C in humidified 5% CO_2_. Plaque titrations were performed in quadruplicates according to Gaush and Smith [21], with minor modifications. Briefly, the virus suspension was serially 10-fold diluted using serum-free DMEM up to 10^−8^, and 250 μl of each dilution was inoculated in corresponding wells of a 24-well plate with 90% confluent MDCK cells. After 1 h of adsorption at room temperature, the inocula were discarded, and the cells were washed with serum-free DMEM and overlaid with DMEM supplemented with 25 mМ HEPES, 0.2% bovine serum albumin, 2 μg/ml of TPCK-trypsin, antibiotics and 1% low melting point agarose (Sigma). The plates were incubated at room temperature until the overlay media became solid, and then they were placed in a 5% CO_2_ humidified atmosphere at 37°C. On day 3 post infection, the monolayers were fixed with a 10% solution of formalin in phosphate buffered saline (PBS, pH 7.4) overnight and plaques were visualised by staining with a 0.05% solution of crystal violet in ddH_2_O. The plaques were counted, and virus concentration was expressed as the number of plaque forming units in one millilitre of suspension (PFU/ml).

### RNA extraction, reverse transcription and PCR

The virus RNA was extracted from 100 μl of allantoic fluid using a RiboPrep RNA extraction kit (Amplisens, Moscow). The RNA pellet was dissolved in 50 μl of RNA dilution buffer, and 10 μl of RNA solution was used to synthesise the cDNA with random hexamers using the Reverta-L kit (Amplisens, Moscow) according to the manufacturer’s manual. The RT reaction mixture was incubated at 37°C for 1 h, followed by 15 min at 70°C. The effectiveness of cDNA synthesis was evaluated by a PCR assay with the primers CU-MF 5` TGATCTTCTTGAAAATTTGCAG 3` and CU-MR 5` TGTTGACAAAATGACCATCG 3` to amplify a 276-bp fragment of the gene for the matrix (M) protein with high sensitivity, as described by Payungporn et al. [22]. The subtype of HA was identified using an H1-H15-specific RT-PCR assay designed by Tsukamoto et al. [23]. Briefly, 15 separate PCR reactions were run with subtype-specific primer pairs. The primers were synthesised commercially according to the previously mentioned report [23]. Each PCR reaction was performed in volume of 25 μl and contained 2.5 μl of cDNA, 2.5 μl of PCR buffer (Sigma), 10 mM dNTP (New England Biolabs), 10 pmol of each primer, 1.5 mM MgSO_4_ and 0.5 μl of DiaTaq DNA polymerase (Amplisens, Moscow). The conditions of PCR included initial denaturation at 94°C for 5 min followed by 35 cycles of denaturation at 94°C for 30 sec, annealing at 50°C for 30 sec and elongation at 72°C for 30 sec. Final elongation was performed at 72°C for 5 min. The resulting PCR products were resolved on a 2% agarose gel with ethidium bromide staining.

### Nucleotide sequencing and phylogenetic analysis

The isolate A/Teal/Tunka/7/2010 has not been previously identified by serological or genetic methods. Therefore, we confirmed the HA subtype identification and identified the NA subtype by phylogenetic analysis of nucleotide sequences of the HA and NA genes in comparison with reference sequences representing all known subtypes of influenza A [24].

The fragments of genes coding for HA and NA were amplified using the procedure developed by Hoffmann et al. [25]. A detailed list of primers used in this study is given in Table 1. The PCR reaction was performed in a volume of 100 μl as described above. The PCR products were resolved on a 0.8% agarose gel, excised from the gel and purified with the QIAgen PCR purification kit (Qiagen). The PCR products were directly sequenced using Sanger’s dideoxy termination method, and obtained nucleotide sequences were deposited in GenBank with accession numbers KF790581 and KF790582 for the HA and NA gene fragments, respectively. Sequences were edited and aligned using BioEdit software.

**Table 1:** Primers used to amplify and sequence the fragments of HA and NA genes of A/Teal/Tunka/7/2010 (H3N8)

**Title**	**Sequence, 5′ - > 3′**	**Target gene**	**References**
H3-919 F	gyatyactccwaatggaagc	HA	Tsukamoto et al. [23]
Bm-NS-890R	atatcgtctcgtattagtagaaacaagggtgtttt	HA	Hoffmann et al. [25]
Bm-NA-1 L	tattggtctcagggagcaaaagcaggagt	NA	Hoffmann et al. [25]
Bm-NA-1413R	atatggtctcgtattagtagaaacaaggagtttttt	NA	Hoffmann et al. [25]

The set of reference sequences included the GenBank accession numbers AF091309 (H1), AY633196 (H2N3), L11129 (H2N9), AY531037 (H3), D90302 (H4), U20460 (H5), AY968676 (H6), U20462 (H7), AB289343 (H8), AY206671 (H9), CY087832 (H10), D90306 (H11), AB288334 (H12), AF250362 (N1), AJ574904, DQ067439 (N2), AY207522, AY207524, AY207513 (N3), CY003986, CY004180 (N4), EU429794 (N5), AY207549 (N6), AB472061, CY014993 (N7), L06587 (N8) and AB292780 (N9). The phylogenetic analysis was performed with the neighbour-joining method; the estimation of evolutionary distances was made based on the Tamura-Nei 93 model of evolution [26], and the significance of the obtained models was evaluated by bootstrap analysis with 1000 replications. The clusters supported by a 70% bootstrap value and higher were assumed to be significant. The Mega 5 program package was used to perform the phylogenetic analysis.

### Evaluation of the toxicity of herb extracts in MDCK cells

MDCK cells were grown to confluence in 96-well culture plates (Sarstedt, USA) and treated in quadruplicate with serial two-fold dilutions of herb extracts in maintenance media, i.e. from 15% to 0.03%. A series of two-fold dilutions of PBS (pH 7.4) in maintenance media was used as the control. Plates were incubated at 37°C in 5% CO_2_ for 7 days. The morphology and viability of cells was evaluated daily using light microscopy. By the seventh day of incubation, cells were washed with sterile PBS (pH 7.4) prewarmed to 37°C, fixed with 10% formalin and stained with crystal violet as described earlier. The stained monolayers were dried, and the stain was extracted with 100 μl of pure methanol. The number of surviving cells was evaluated by measuring the optical density of the corresponding extract at a wavelength of 630 nm using an Immunochem 2100 microplate spectrophotometer (High Technology Inc., USA).

### Virus neutralisation test

MDCK cells were grown to confluence in 24-well plates. Replicates of AIV A/Teal/Tunka/7/2010 (H3N8) were diluted in culture media without foetal calf serum to achieve a virus concentration of approximately 1 × 10^5^ PFU in 500 μl. Then, 500 μl of the virus suspension was mixed with 500 μl of freshly prepared 2% extracts of *G. decumbens*, *H. erectum*, *P. bistorta*, *and* Deva-5 or 1% extracts of *T. chebula* and *M. cochinchinensis*. Specific anti-HA antibodies to homologous H3 subtype of influenza A virus were used as the positive control for neutralisation. The antibodies were bought as a commercial antiserum against influenza virus H3N2 (Research Institute of Influenza, Saint Petersburg, Russian Federation). The antiserum was diluted 1:10 in sterile ddH_2_O, and 100 μl of diluted serum was mixed with 400 μl of maintenance media and 500 μl of the virus suspension. The negative control was prepared by mixing 500 μl of sterile ddH_2_O with 500 μl of the virus suspension. The mixtures were incubated at 37°C for 30 min, and then the concentration of infectious virus in each sample was established using plaque assays. The neutralisation index was estimated as the quotient of the AIV concentration in the negative control to the AIV concentration in the test sample. This test was performed with six independent repeats.

### Plaque reduction neutralisation test (PRNT)

The 2% extracts of *G. decumbens*, *H. erectum*, *P. bistorta* and Deva-5 or 1% extracts of *T. chebula* and *M. cochinchinensis* were serially diluted two-fold to a concentration of 0.016% in DMEM media supplemented with 25 mМ HEPES, 0.2% bovine serum albumin, 2 μg/ml of TPCK-trypsin and antibiotics. Sterile ddH_2_O and the specific antihaemagglutin antibodies to the homologous H3 subtype of Influenza A virus were used as controls as described above. Each dilution of the extracts and controls was supplemented with approximately 200 PFU of A/Teal/Tunka/7/2010 (H3N8) virus and incubated at 37°C for 30 min. Afterwards, each sample was placed into the wells of 12- or 24-well plates with a 90% confluent monolayer of MDCK cells. Adsorption was conducted at room temperature for 60 min, and then the inocula were discarded and monolayers were covered with overlay media for the plaque assay. At day 3 post infection, the cells were fixed, and the plaques were visualised with crystal violet and counted. Four independent repeats were done for plaque reduction neutralisation test. The 50% and 90% inhibition doses were estimated for each extract as the maximal reciprocal dilution of extract that inhibited plaque formation by 50% or 90% (ID50% and ID90%, respectively).

### Data analysis

Results are presented as the mean value of at least four independent repeats. The standard deviation of the mean was used to evaluate the variability of data. The Kolmogorov-Smirnof test was used to check the normality of the sample, and Student’s t-test was used to evaluate differences between samples. A value of p ≤ 0.05 was considered statistically significant. The ID50% and ID90% were estimated using probit analysis. The relationship between the concentration of extracts and infectivity of the treated virus was evaluated using the Pearson correlation test. The correlation was assumed to be significant at R > 0.7 (p = 0.05). Data analysis was performed using Statistica 6.1 and MSOffice EXCEL 2003 software.

## Results

### Virus subtype and culture properties

**Figure 1:**
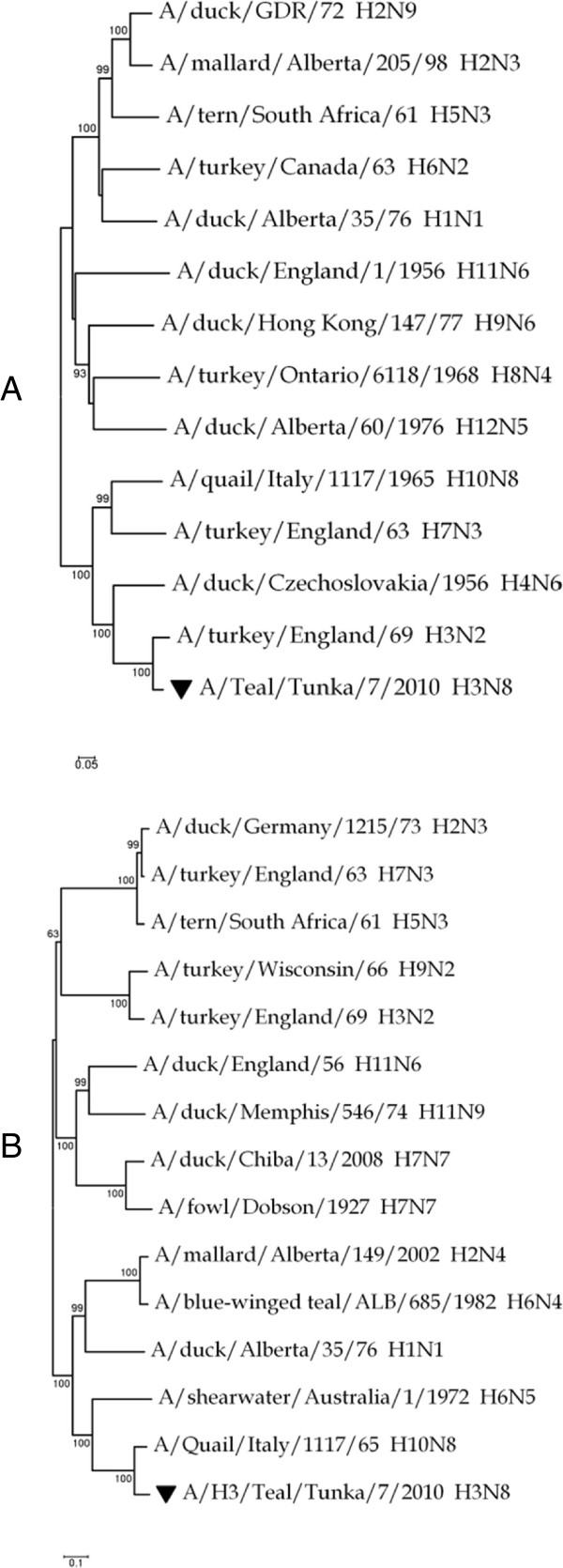
**Identification of subtype of influenza A virus isolate A/Teal/Tunka/7/2010. A** – phylogenetic analysis of a 714 bp fragment of the HA gene; **B** – phylogenetic analysis of a 1216 bp fragment of the NA gene. The sequences of A/Teal/Tunka/7/2010 are labelled with inverted triangles. The phylogenetic analysis was performed using the neighbour-joining method; the estimation of evolutionary distances was made on the basis of the Tamura-Nei 93 model of evolution Tamura and Nei [26]. The significance of trees was evaluated by bootstrap analysis with 1000 replications.

The isolate A/Teal/Tunka/7/2010 (H3N8) caused a clear cytopathic effect in MDCK cells on day 3 post infection. At this time, it produced small (0.3–0.5 mm) plaques and reached a titre of 1 ± 0.2 × 10^6^ PFU/ml. The subtype-specific RT-PCR produced a single band with primers corresponding to the HA subtype H3. The phylogenetic analysis of the fragments of HA and NA genes had shown that isolate A/Teal/Tunka/7/2010 (H3N8) has HA of the H3 subtype and NA of the N8 subtype (Figure 1).

### Plant extracts are toxic for MDCK cells at concentrations higher than 2%

**Figure 2:**
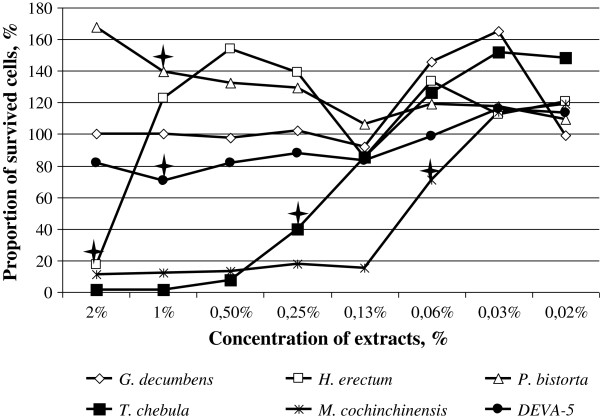
**Viability of MDCK cells (%) treated with different concentrations of plant extracts.** The lowest concentration at which toxic (or enhancing) effects reach statistical significance (p < 0.05) are labelled by quadrangular stars.

All plant extracts were toxic to MDCK cells at concentrations higher than 2%. The most toxic extracts were derived from *T. chebula* and *M. cochinchinensis*, causing changes in cell morphology at concentrations as low as 0.06%. Plant extracts from *P. bistorta* and the Deva-5 composition were less toxic, as single MDCK cells were detected under the light microscope when the culture media contained up to 7.5% of those preparations, although no cells survived at higher concentrations. The results of the cell viability assay at concentrations of plant extracts less than 2% are presented in Figure 2.

The cytotoxic effect was dose-dependent, and the plant extracts had different cytotoxic profiles. Thus, the highly toxic extracts of *T. chebula* and *M. cochinchinensis* killed up to 100% of cells, except for the very low concentrations of 0.5% and 0.13%, respectively. At higher dilutions, these preparations caused a dose-dependent increase in cell viability and became non-toxic at concentrations of 0.13% and 0.03%, respectively. The extracts of *G. decumbens*, *P. bistorta* and Deva-5 expressed weak cytopathic effects and did not cause significant damage to cells at concentrations of 2% and less. The extracts of *H. erectum* destroyed 80% of cells at 2%, but after being diluted only twice, they caused no significant cytopathic effects. These results suggest that extracts of *G. decumbens*, *H. erectum, P. bistorta* and Deva-5 have no significant toxicity at concentrations of 2% and less, whereas extracts of *T. chebula* and *M. cochinchinensis* were well-tolerated by MDCK cells at concentrations 0.13% and 0.03%, respectively.

### Virus neutralisation by plant extracts

**Figure 3:**
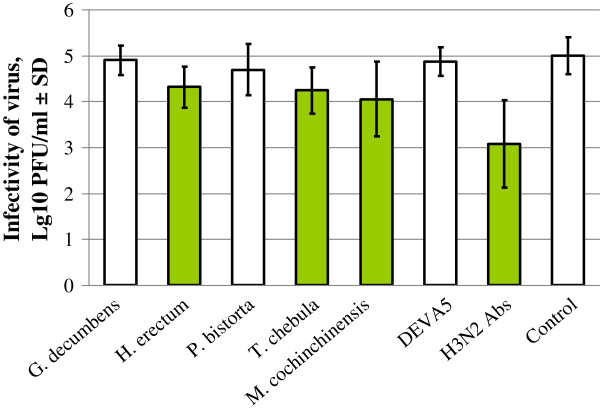
**Titres of infectious virus in samples containing ~1 × 10**^**5 **^**PFU A/Teal/Tunka/7/2010 (H3N8) treated with 1% plant extracts.** Control – water-treated virus; H3N2 Abs - A/Teal/Tunka/7/2010 (H3N8) treated with 1:100 diluted specific antibodies to the H3N2 influenza virus. Green blocks designate the samples that showed a significant reduction in viral titres in comparison to the control by Student’s t-test (p ≤ 0.05). Error bars reflect standard deviations.

Treatment of high doses of virus with high concentrations of plant extracts did not affect the infectivity of virus, with the exception of extracts of *H. erectum*, *M. cochinchinensis* and *T. chebula*. These three extracts significantly reduced the infectivity of A/Teal/Tunka/7/2010 (H3N8) by approximately five-fold (Figure 3). The specific antibodies reduced virus infectivity by 100-fold, which indicates the weak neutralising ability of these plant extracts.

**Figure 4:**
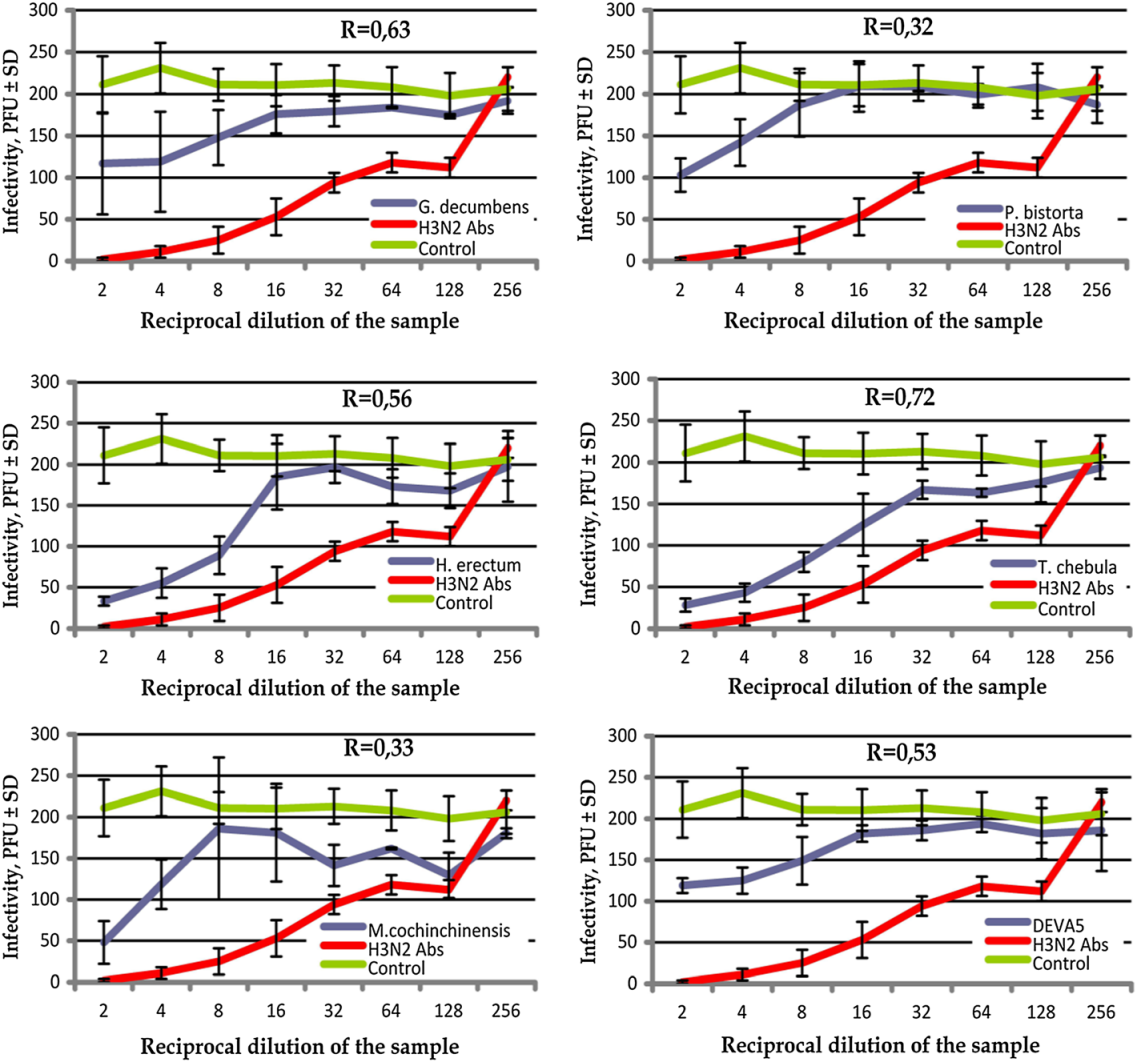
**Neutralising activity of different concentrations of plant extracts against influenza A virus A/Teal/Tunka/7/2010 (H3N8).** Red line – specific antibodies to the H3 subtype of influenza A virus; green – non-treated virus; blue – virus treated with the corresponding plant extract. The data reflect the mean values of four independent repeats of the PRNT assay as described in the Methods; error bars show standard deviations. The correlation coefficients R between the reciprocal dilution of extracts and infectivity of A/Teal/Tunka/7/2010 (H3N8) are shown in each panel. The correlation was assumed to be statistically significant at R > 0.7 (p = 0.05).

The plaque reduction neutralisation tests revealed that none of the extracts were able to inhibit plaque formation by 90%. However, three extracts, i.e. *H. erectum, T. chebula* and *M. cochinchinensis*, inhibited plaque formation by more than 50% at low dilutions, i.e. from 1:3 to 1:14 (Table 2). Specific antibodies to the H3 subtype influenza virus inhibited plaque formation by 90% at a dilution of 1:6 (ID90% = 6) and plaque formation by 50% at a dilution of 1:60 (ID50% = 59.1). Only one preparation, *T. chebula*, exhibited a significant positive correlation (R > 0.7 at p = 0.05) between the dilution factor of the extract and the infectivity of the virus (Figure 4). These results are summarised in Table 2 where the neutralisation indexes and 50% inhibition doses are given.

**Table 2:** Direct antiviral action of plant extracts against influenza A virus A/Teal/Tunka/7/2010 (H3N8)

**Extract**	NI ± SD^*^	ID50%^**^
*Gentiana decumbens*, 1% extract	1.4 ± 0.7	-
*Hypecoum erectum*, 1% extract	4.8 ± 0.2	8.6
*Polygonum bistorta*, 1% extract	1.6 ± 0.1	-
*Terminalia chebula*, 0.5% extract	4.9 ± 0.2	13.4
*Momordica cochinchinensis*, 0.5% extract	5.1 ± 0.3	2.7
DEVA-5, 1% extract	1.6 ± 0.4	-
H3N2 Abs, diluted as 1:100	93.9 ± 0.7	59.1

* - approximately 1 × 10^5^ PFU of A/Teal/Tunka/7/2010 (H3N8) were used for the NI assay.

** - approximately 200 PFU of A/Teal/Tunka/7/2010 (H3N8) were used for the PRNT assay.

The neutralisation index (NI) was estimated as the quotient of the virus concentration in the water-treated sample to the virus concentration in the test sample. The 50% inhibition dose was estimated using probit analysis as the maximal reciprocal dilution of extract that inhibited the formation of plaques by 50%.

## Discussion

Deva-5 is used in traditional medicine to treat infectious diseases, including seasonal and epidemic flu. *G. decumbens* and *H. erectum* have been described as antimicrobial herbs in books of traditional medicine [9]. In this study, we tested the antiviral activities of the traditional drug Deva-5 and its components against avian influenza virus H3N8 in cell culture. The virus strain A/Teal/Tunka/7/2010 (H3N8) used in our study was previously characterised in a very restricted set of experiments. Therefore, we sequenced the HA and NA genes to exclude possible misidentification of the virus subtype or the misplacement of virus stock. The genetic identification of the virus subtype was performed by phylogenetic analysis in comparison with reference strains of each subtype and confirmed the virus identity.

Although Deva-5 did not show concentration-dependent antiviral activity, some signs of virus inhibition by Deva-5 were observed at the 1% concentration of the extract (Figure 4). This effect can be explained by the presence of three components that consistently reduced the infectivity of A/Teal/Tunka/7/2010 (H3N8): *H. erectum* and *T. chebula* and *M. cochinchinensis*. These three extracts inhibited the virus much more weakly than specific anti-HA antibodies; however, this may have been due to the very low concentrations of bioactive substances in the water extracts. For example, in similar research on *Scutellaria baicalensis*, the authors used 30 g of a crude powder treated with 200 ml of various organic solvents. Afterwards, the preparations were freeze-dried, and working solutions with concentrations of extract equal to 1 mg/ml were prepared. Under these conditions, the IC_50_ in PRNT against IAV H1N1 varied from 14 μg/ml to 134 μg/ml [27]; this approximately corresponds to a reciprocal dilution from 10 to 100. In our research, we used only 1–2 grams of homogenised herbs or seeds, and in the PRNT these extracts exhibited an IC_50_ equal to a reciprocal dilution between 8 and 14. So, it is likely that, if the concentration of biologically active components had been increased (e.g. by freeze-drying, or with chemical fractionation to remove toxic substances), the antiviral properties would have become stronger as well. Thus, although Deva-5 and its constituents have extremely low antiviral activity, if any, the extracts of *H. erectum, T. chebula* and *M. cochinchinensis* are promising potential sources of new antiviral drugs.

Deva-5 exhibited weak cytotoxicity and did not cause significant damage to MDCK cells at concentrations less than 2%. The possible rationale for the use of Deva-5 instead of separate plant extracts is the cumulative effect of the action of its constituents. Indeed, the extracts of *H. erectum, T. chebula* and *M. cochinchinensis* exhibited antiviral action, but were also quite toxic to mammalian cells (Figure 2). In contrast, the extracts of *G. decumbens* and *P. bistorta* exhibited no antiviral action, but possessed very low cytotoxicity. Moreover, an approximate increase of 60% in the cell number in samples treated with extracts of *P. bistorta* suggests that this constituent may have a stimulating effect on mammalian cell growth (Figure 2). Thus, Deva-5 consists of two types of components: “antivirals” that exert inhibition of the virus but also destroy mammalian cells; and “cell protectors” that compensate for the negative effects of antiviral constituents.

It has been previously reported that the *H. erectum* plant contains steroidal saponins, alkaloids, and coumarins [14]. However, no antiviral substances have been isolated from this plant. Likewise, no reports have been published on antiviral constituents isolated from *M. cochinchinensis*. This plant was shown to contain momorchochin, alkenes, fatty acids, carotenoids, triterpenoidal saponins, cochinin B and a specific chymotripsin inhibitor. Three other plants have been reported to contain phenolic compounds and flavonoids that possess strong anti-influenza viral activity [12]. In addition *G. decumbens* contains secoiridoids and alkaloids, *P. bistorta* contains fatty acids, steroids and triterpenoids, and *T. chebula* contains fatty acids, fructose, amino acids, anthraquinone and triterpenoids [17]. The protective activity of *T. chebula* against influenza A virus (H1N1) has been reported; however, in contrast to our findings, no direct antiviral activity of the water extract of *T. chebula* was detected in this study [19]. This discrepancy may have been caused by the different subtype of IAV used in our study (H3N8). In this case, the antiviral substances of *T. chebula* might have a subtype-specific inhibitory activity against IAV. Further research is necessary to resolve this question. Thus, for the first time, the direct antiviral action of two components of the Deva-5 herb formulation, *H. erectum* and *T. chebula*, was demonstrated in this work*.*

Our preliminary study showed that Deva-5 inhibited the growth of *Staphylococcus aureus*. Hexane and dichloromethane extracts from *M. cochinchinensis* leaves, as well as seven alkaloids isolated from *H. erectum*, have antimicrobial activity against a number of Gram-negative and Gram-positive bacteria, including *Staphylococcus aureus*[28,14]. Bag et al. showed that a hot aqueous extract of *T. chebula* fruit was found to be potent against *Staphylococcus aureus* strains, whereas an ethanol extract was found to be more potent against *Escherichia coli* strains [18]. Also, an aqueous extract of *T. chebula* exhibited antifungal activity against a number of dermatophytes and pathogenic yeasts and the acetone extract of *T. chebula* seeds showed antiplasmodial activity against *Plasmodium falciparum*[18]. Taken together, these results suggest that rationality of the use of Deva-5 in traditional medicine to treat infectious diseases can be explained by the antiviral and antimicrobial properties of its components.

## Conclusions

For the first time, the consistent direct antiviral action of extracts of *H. erectum, T. chebula* and *M. cochinchinensis* was demonstrated. These extracts significantly reduced the infectiveness of influenza A virus H3N8 in vitro when used at high concentrations (0.25–1%). However, Deva-5 itself and the remainder of its components did not exhibit significant antiviral activity. These results suggest that the former three plants contain substances with significant antiviral activity and could be a promising source of new antiviral drugs. However, further research, including in vivo tests in an animal model, is necessary to identify the biologically active components and to remove toxic substances.

## Abbreviations

IAV: Influenza A virus; AIV: Avian influenza virus; HA: Haemagglutinin; NA: Neuraminidase.

## Competing interests

The authors declare that they have no competing interests.

## Authors’ contributions

NO conceived the study, performed the preparation of extracts, participated in study design, participated in data analysis and interpretation and wrote the manuscript; MAK designed the study, performed the virological experiments, participated in the molecular genetic studies, carried out data analysis and interpretation and wrote the manuscript; AVL carried out the molecular, genetic and virological studies, participated in data analysis and critically discussed the manuscript; GAD, JO, CC, SO, PM and JB participated in study design and coordination and helped to interpret the results and draft the manuscript. All authors contributed to manuscript preparation and approved the final manuscript.

## Pre-publication history

The pre-publication history for this paper can be accessed here:

http://www.biomedcentral.com/1472-6882/14/235/prepub
